# Surgical management of native aortic valve leaflet avulsion during TAVR

**DOI:** 10.1186/s13019-024-02973-8

**Published:** 2024-07-30

**Authors:** David M. Williams, Andrew Castellano, William Phillips, Kelly Miller, Aakash Garg

**Affiliations:** 1 Department of Cardiothoracic Surgery, Ellis Medicine / St. Peter’s Health Partners, 1201 Nott Street Suite 307, Schenectady, NY 12308 USA; 2 Cardiology Associates of Schenectady, St. Peter’s Health Partners, 2546 Balltown Road Suite 300, Schenectady, NY 12309 USA

**Keywords:** TAVR, Leaflet avulsion, Structural heart, Balloon valvuloplasty

## Abstract

**Background:**

Transcatheter aortic valve replacement (TAVR) has increased in utilization since its approval for management of aortic stenosis patients across all risk strata. We report a rare case of aortic valve leaflet avulsion after balloon expandable TAVR managed with urgent surgery.

**Case presentation:**

A 78-year-old male underwent TAVR complicated by avulsion of the native aortic valve leaflet’s left coronary cusp. He was taken for urgent surgery for cusp resection to prevent thromboembolic complications.

**Conclusions:**

Native aortic valve leaflet avulsion should be suspected during TAVR in instances of extreme hemodynamic instability after balloon aortic valvuloplasty. As TAVR expands in lower risk patients, surgeons should have a low threshold to intervene surgically to treat native leaflet avulsion or other complications.

**Supplementary Information:**

The online version contains supplementary material available at 10.1186/s13019-024-02973-8.

## Background

Transcatheter aortic valve replacement (TAVR) has revolutionized the treatment of severe aortic stenosis and is now approved for patients of all surgical risk groups [1]. Commonly recognized complications of TAVR include the need for permanent pacemaker implant, stroke, bleeding, and vascular injury at the access site [2]. We present herein a rare case of native aortic valve leaflet avulsion during TAVR that was treated with urgent surgery.

## Case presentation

**Fig. 1:**
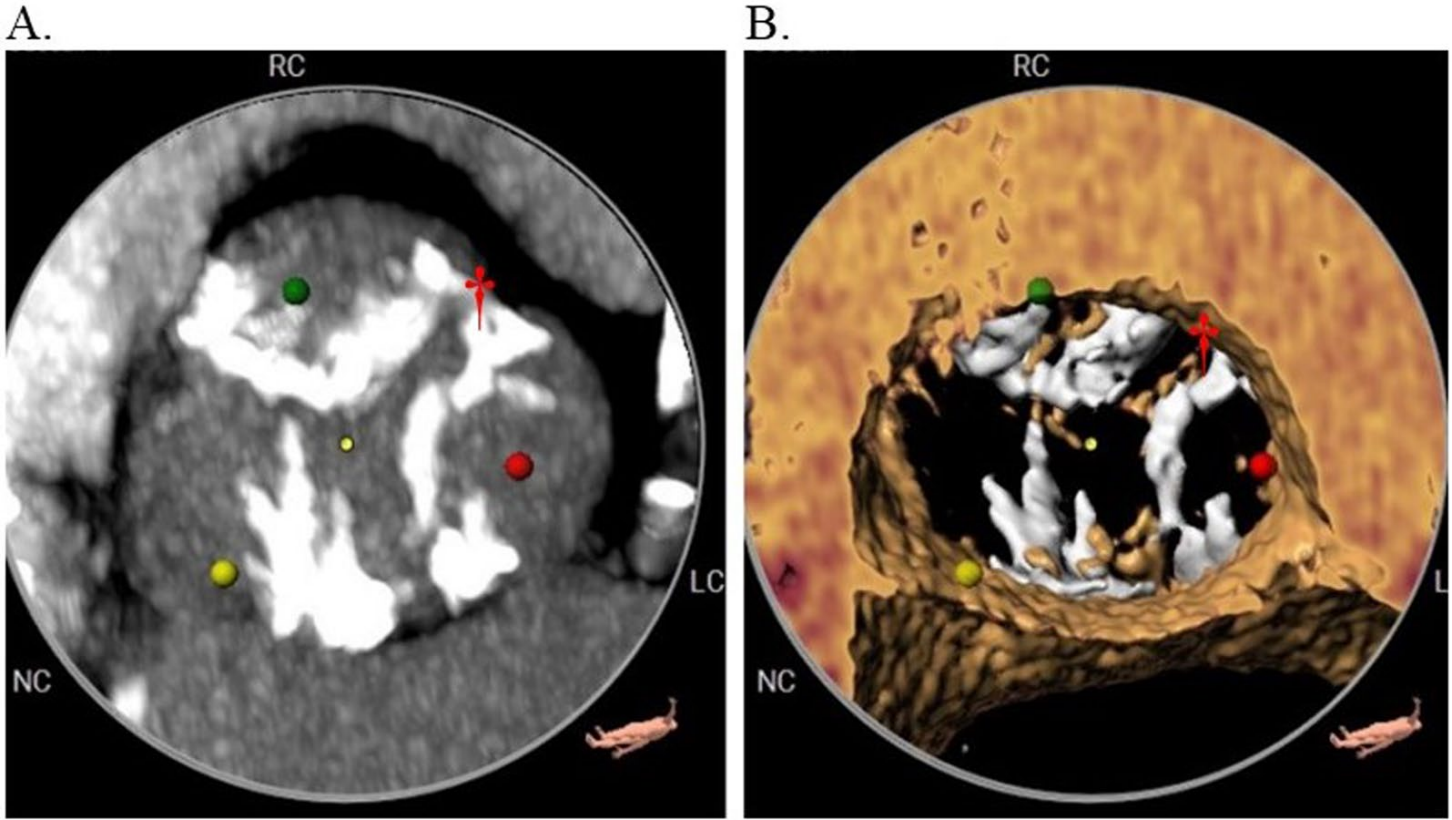
Preoperative ECG-gated computed tomographic images of trileaflet aortic valve stenosis. Heavy calcification of all three leaflets can be seen on both the maximum intensity projection (**A**) and augmented reality (**B**) images. The left coronary cusp (red dot) was avulsed during TAVR and found to be anchored at the commissure of the left and right coronary cusps (†) during surgical exploration

A 78-year-old male, who gave consent to disclose the information included in this report, was referred for management of symptomatic critical aortic stenosis (EF 65%, mean gradient 94 mmHg, aortic valve area 0.52 cm^2^). He was evaluated by our Heart Team and was deemed intermediate risk given his age, frailty, and functional status (Society of Thoracic Surgeons mortality risk 2.1%). Further workup including an ECG-gated computed tomographic scan with TAVR protocol demonstrated a heavily calcified trileaflet aortic valve and acceptable transfemoral access for TAVR (Fig. 1).

After advancement of large bore sheath via percutaneous right femoral access, heparin was administered to an activated clotting time of 268 s. The aortic valve was crossed with a 0.035” Emerald™ Guidewire (Cordis, Florida, USA) and a 5Fr DxTerity™ AL1.0 catheter (Medtronic, Minnesota, USA). There was some initial difficulty getting the wire to cross the aortic valve. The AL1 catheter and pre-dilation balloon passed through the valve easily without resistance. The native aortic valve was pre-dilated with an 18 mm Z-MED™ balloon (B. Braun Medical, Pennsylvania, USA) after which transient severe hypotension was noted. A 26 mm balloon expandable SAPIEN 3 prosthesis (Edwards Lifesciences, California, USA) was implanted with immediate hemodynamic improvement.

**Fig. 2:**
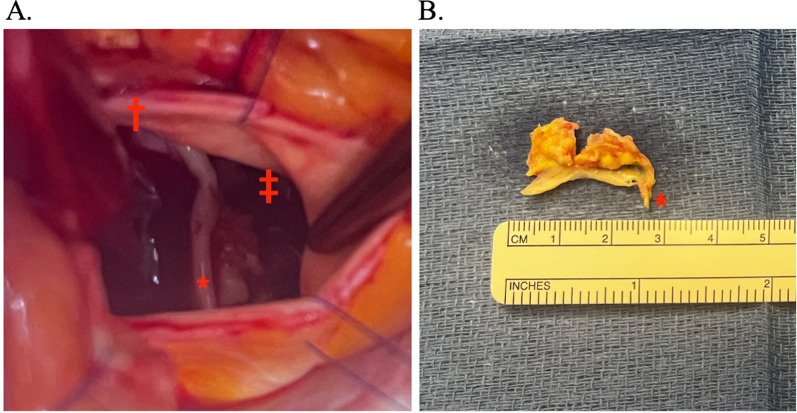
Surgical view of native aortic valve leaflet avulsion after balloon expandable TAVR. **A** Intraoperative image of the avulsed left coronary cusp in situ (*) with attachment near the left/right commissure (†). Reflections of the prosthesis cage are seen towards the right (‡). **B** Excised left aortic valve cusp showing tissue (*) which had been anchoring the valve cusp to the left/right commissure before surgical excision

Post-procedure transthoracic echocardiography demonstrated normal functioning of the TAVR prosthesis, however, a highly mobile linear echodensity was noted just above the aortic valve prosthesis. An urgent transesophageal echocardiograph confirmed a 1.6 cm echodensity with possible attachment to the prosthetic valve (Video 1). At this point, our differential diagnosis was early prosthesis thrombus versus native leaflet avulsion. We therefore returned emergently to the hybrid operating room for attempted percutaneous thrombectomy with the Indigo^®^ Aspiration System (Penumbra Incorporated, California, USA). Close inspection of fluoroscopy during the attempted thrombectomy revealed a highly mobile calcified density near the left coronary cusp thus raising our suspicion of avulsed native aortic valve leaflet (Video 2). The thrombectomy was therefore aborted and after discussion with patient, the decision was made for surgical intervention. After central cannulation for cardiopulmonary bypass, an aortotomy was made just distal to the palpable transcatheter aortic valve. The entire left coronary cusp of the aortic valve had been avulsed from its annulus and was held in place by a small piece of aortic intima at the commissure between the left and right coronary cusps (Fig. 2). The avulsed leaflet was resected and a saline test confirmed competency of the prosthesis. The patient was discharged without further events and was doing well at 6-month follow-up.

## Discussion and conclusions

There are two key takeaways from this case. First, TAVR operators should be aware of aortic valve leaflet avulsion and the methods to identify it. It should be suspected in cases of severe hemodynamic instability and transient torrential aortic insufficiency immediately between balloon aortic valvuloplasty and TAVR valve deployment, especially if there is initial difficulty crossing the aortic valve [3]. It has been our experience that crossing critically stenosed aortic valves is often challenging. We attribute this to the diminutive aortic valve area creating a small target for the wire and the high velocity jet pushing the wire away from the valve orifice. In this case, however, the wire presumably crossed through a non-calcified portion of the left coronary cusp body near the annulus. There was no indication of a defect of this cusp on preoperative workup. Avoiding aortic valve leaflet avulsion can thus be best achieved by ensuring the wire crosses through the aortic valve orifice. To avoid this complication in the future, we recommend removing the wire and recrossing the valve if there was any difficulty with the initial crossing or the wire does not settle in expected location based on the valve’s calcification pattern. Aortic valve leaflet avulsion can be differentiated from other causes of hemodynamic collapse during TAVR. Unless there is significant residual aortic insufficiency, which can be quickly ruled out with an aortogram, hemodynamics should normalize immediately upon delivery of the aortic valve prosthesis in aortic valve leaflet avulsion. In contrast, there will be no changes after valve deployment in other causes of hemodynamic collapse such as pericardial tamponade or aortic dissection. If leaflet avulsion is suspected, careful fluoroscopic inspection can be useful to diagnosis the problem as is evident in our case and others [4].

The second key takeaway from this case is that it is important for interventionalists and surgeons to understand and employ the various treatment options for native leaflet avulsion. Because TAVRs have historically been performed in high or prohibitive surgical risk patients, the rare instance of native aortic valve leaflet avulsion has been previously treated with observation, anticoagulation, percutaneous snare retrieval, or a combination of these approaches [4–6]. Unlike previous reports, we chose not to snare the avulsed leaflet because of its large size, the patient’s acceptable surgical risk, and inherent risk of damaging the aortic intima by placing undue traction on the avulsed leaflet. As TAVR expands in the younger and low surgical risk populations, it is important for operators to have a low threshold for surgical intervention in the case of native aortic valve leaflet avulsion or other complications.

### Supplementary Information

Supplementary Material 1.

Supplementary Material 2.

## Data Availability

All data generated or analyzed during this study are included in this published article.

## Abbreviations

TAVR: Transcatheter aortic valve replacement
ECG: Electrocardiogram
